# Nuclear DNA content in Miscanthus sp. and the geographical variation pattern in *Miscanthus lutarioriparius*

**DOI:** 10.1038/srep34342

**Published:** 2016-10-04

**Authors:** Jiajing Sheng, Xiaohu Hu, Xiaofei Zeng, Ye Li, Fasong Zhou, Zhongli Hu, Surong Jin, Ying Diao

**Affiliations:** 1 State Key Laboratory of Hybrid Rice, College of Life Science, Wuhan University, Wuhan 430072, China; 2 School of Chemistry, Chemical Engineering and Life Sciences, Wuhan University of Technology, Wuhan 430070, China

## Abstract

The genome sizes of five *Miscanthus* species, including 79 accessions of *M. lutarioriparius*, 8 of *M. floridulus*, 6 of *M. sacchariflorus*, 7 of *M. sinensis*, and 4 of *M.* × *giganteus* were examined using flow cytometry. The overall average nuclear DNA content were 4.256 ± 0.6 pg/2C in *M. lutarioriparius*, 5.175 ± 0.3 pg/2C in *M. floridulus*, 3.956 ± 0.2 pg/2C in *M. sacchariflorus*, 5.272 ± 0.2 pg/2C in *M. sinensis*, and 6.932 ± 0.1 pg/2C in *M.* × *giganteus*. Interspecific variation was found at the diploid level, suggesting that DNA content might be a parameter that can be used to differentiate the species. Tetraploid populations were found in *M. lutarioriparius, M. sacchariflorus*, and *M. sinensis*, and their DNA content were 8.34 ± 1.2, 8.52, and 8.355 pg, respectively. The association between the DNA content of *M. lutarioriparius*, collected from representative ranges across the Yangtze River, and its geographic distribution was statistically analyzed. A consistent pattern of DNA content variation in 79 *M. lutarioriparius* accessions across its entire geographic range was found in this study. Along the Yangtze River, the DNA content of *M. lutarioriparius* tended to increase from the upstream to the downstream areas, and almost all tetraploids gathered in the upstream area extended to coastal regions.

*Miscanthus* is a tall (−5 m), rhizomatous, and perennial grass genus. It is primarily native to a wide range of climates from eastern Asia south to the Pacific islands^1^. It has recently been thoroughly investigated as a promising bioenergy candidate because of its high biomass yield potential, stress tolerance and photosynthetic efficiency^2^. The genetic diversity of *Miscanthus* in different temperate latitudes and their tolerance of varying environmental conditions are a key feature of interest. Some *Miscanthus* genotypes are cold tolerant and maintain high photosynthetic rates at temperatures below 12 °C, whereas sugarcane, maize, and sorghum show significantly reduced CO_2_ assimilation at temperatures below 14 °C^3^,^4^. Their adaptation to different temperate climates can be exploited to study their genetic diversity. China is the center of the geographical distribution of *Miscanthus*; therefore, it has greater plant diversity and numerous ecotypes^5^. *Miscanthus lutarioriparius* L.Liou ex S.L. Chen & Renvoize (formerly called *Triarrhena lutarioriparia*) is endemic to China and grows on lakesides and flooded river banks south of the middle and lower reaches of the Yangtze River. These abundant germplasm resources could be used to analyze the intraspecific DNA content variation at the taxonomic level, the evolution of the genus, and the influence of environmental diversity on genome size. These data can provide valuable information for selective breeding and biomass crop improvement programs, thereby producing cultivars with higher yields, broader adaptability, and better quality.

*Miscanthus* is a promising alternative bioenergy crop, and its yield, biomass properties, morphology, phylogenetic relationships, polyploidy induction, and genetic variation based on Simple Sequence Repeats (SSRs) have been studied^6^,^7^,^8^,^9^,^10^. Previously, the approximate nuclear DNA content of several diploid *Miscanthus* species was reported to be approximately 4.37 ± 0.02 pg/2C in *M. lutarioriparius*, 5.2 ± 0.2 pg/2C in *M. sinensis.* Anderss, 5.1 ± 0.2 pg/2C in *M. floridulus* (Lab) Warb. exSchum. etLaut, 4.3 ± 0.2 pg/2C in *M. sacchariflorus.* Nakai, and 7.0 pg/2C in triploid *M.* × *giganteus*^10^,^11^,^12^. However, in previous evaluations of the *Miscanthus* nuclear DNA content, only a limited number of accessions were included, and little is known regarding the variation in its genome size across its distribution range. Hence, detailed research that focuses on a quantitative classification analysis of interspecific variation (between five species) and intraspecific variation (in one species) along its geographic distribution should be performed.

Nuclear DNA content is a basic characteristic of a species that can be used for a wide range of biological investigations, including hybrid identification, taxonomy, and evolutionary studies^11^,^13^. Information on DNA content is very effective for the genomic characterization of species; this information provides data for comparative studies in a variety of taxon and provides insight into how genomes vary during evolution^14^. Furthermore, comparative analysis of the *Miscanthus* nuclear DNA content among species that are distributed in different geographical environments can be used to explore their evolution and adaptability to changing environments. Flow cytometry is useful in assessing genome size, ploidy level, and for screening populations in detail^15^. It can also be efficiently applied to population biology, crop breeding, and quality control in commercial seed production for the success of breeding programs^13^.

A survey of *M. lutarioriparius* nuclear DNA content variation across its geographic distribution is crucial to obtain a better understanding of the evolutionary origin of polyploids, the patterns of genome growth or shrinkage across China and the environmental adaptation of this species. Knowledge of genetic variation also provides important information for *Miscanthus* breeding programs with respect to the long-term improvement of biomass yield performance. This study explored the dominant *Miscanthus* species distribution in natural populations and the intraspecific variation in *M. lutarioriparius, M. sinensis*, and *M. floridulus* based on nuclear DNA content. Our objectives were as follows: (1) to explore the differences among five species of *Miscanthus* based on nuclear DNA content; (2) to determine whether there is any intra-ploidy genome size variation in *M. lutarioriparius*; and (3) to determine the geographical patterns of *M. lutarioriparius* based on nuclear DNA content, thereby more precisely defining the diploid and tetraploid locations.

## Results

### Determining the nuclear DNA content of *M. lutarioriparius, M. floridulus, M. sacchariflorus, M. sinensis*, and *M.* × *giganteus*

**Figure 1:**
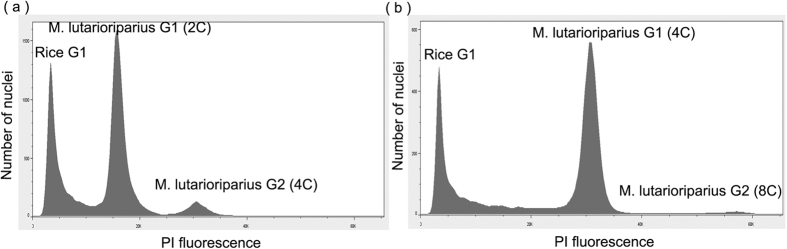
Flow cytometric histogram of the diploid *M. lutarioriparius* (**a**) and the tetraploid *M. lutarioriparius* (**b**) stained with Propidium Iodide and mixed with rice nuclei (internal standard).

The flow cytometric histogram revealed three distinct peaks (Fig. 1). The first peak was the G1 peak of the internal control. The next two peaks represented the G1 and G2 peaks of *Miscanthus sp*. The DNA content of diploid *M. lutarioriparius* (Fig. 1a), *M. sacchariflorus, M. floridulus, M. sinensis*, and triploid *M.* × *giganteus* was estimated to be 4.26 ± 0.6 pg/2C, 3.96 ± 0.2 pg/2C, 5.17 ± 0.3 pg/2C, 5.27 ± 0.2 pg/2C, and 6.93 ± 0.1 pg/2C, respectively. *Miscanthus lutarioriparius, M. sacchariflorus*, and *M. sinensis* had tetraploid accessions, with average DNA content of 8.34 ± 1.2, 8.42, and 8.355 pg, respectively. The detailed average nuclear DNA content for the 104 individual plants of *Miscanthus* is reported in Supplementary Tables S1–2.

### Analyzing the interspecific genetic variance in *M. lutarioriparius, M. floridulus, M. sacchariflorus*, and *M. sinensis*

Student-Newman-Keuls (SNK) multiple comparison tests were conducted to analyze the correlations and variations among the four species (Table 1). From the analyses, the four species were distinct and were divided into three subsets. More specifically, *M. floridulus* and *M. sinensis* were classified together under set 3, whereas the other two species were divided into sets 1 and 2. *Miscanthus floridulus* and *M. sinensis* had significant differences from the other two species. *Miscanthus sacchariflorus* had the lowest nuclear DNA content. *M. floridulus* and *M. sinensis* had similar genome sizes and were closely related.

### Analyzing intraspecific genetic diversity in *M. lutarioriparius*

**Figure 2:**
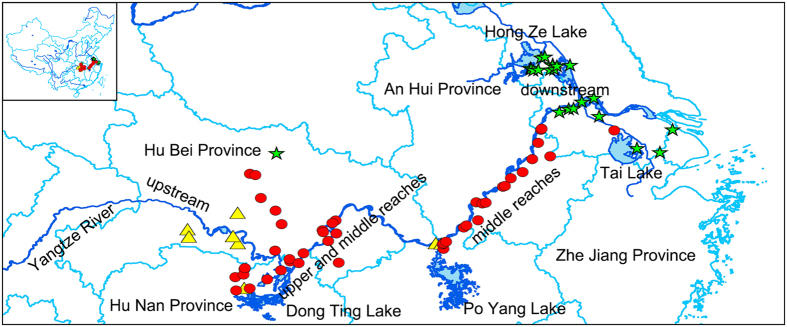
The geographical distribution of *M. lutarioriparius* in 79 populations across the study area and the major river basin and lake along Yangtze River (blue line). The yellow triangles: diploid plants with genome sizes less than 4.0 pg; the green star like stars: tetraploid plants; the red spotsdots: diploid plants and genome sizes bigger than 4.0 pg. The maps were drawn by ESRI ArcMap 10.0 (http://www.esri.com).

**Figure 3:**
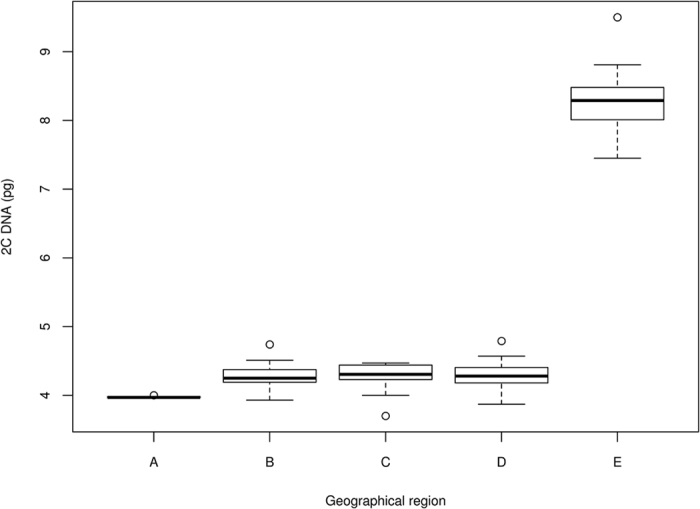
Box-plot graph of 2C nuclear DNA contents in *M. lutarioriparius* from five geographic regions. 1: upstream Hubei Province; 2: upper and middle reaches Huna Province; 3: upper and middle reaches Hubei Province; 4: middle reaches Anhui Province; 5: downstream Jiangsu Province. Horizontal line represent the median, boxes span the interquartile range, and whiskers the non-outlier ranges. Circles denote outliers. The figure was drawn by R version 3.0.2.

All 79 individuals representing native Chinese populations of *M. lutarioriparius* were divided into the following five groups based on their geographical distributions: upstream Hubei Province, upper and middle reaches Hunan Province, upper and middle reaches Hubei Province, middle reaches Anhui Province and downstream Jiangsu Province (Fig. 2). Statistical analyses revealed significant differences in the DNA contents among the five populations and clear genetic boundaries among these populations (one-way ANOVA, P < 0.001, Table 2). A Student-Newman-Keuls (SNK) test indicated that the five populations of *M. lutarioriparius* could be divided into the following three subsets (Table 2): the smaller nuclear DNA content (below 4.0 pg), intermediate diploid DNA content (4.0 pg to 4.8 pg), and tetraploid DNA content (8.10 pg). The three subsets were consistent with the geographical distribution, and nearly all tetraploid *M. lutarioriparius* were grouped in the upper reaches (Fig. 2). *Miscanthus lutarioriparius* inhabiting the downstream area contained the largest genomes, whereas those in the upper reaches had smaller genomes. A maximum intrapopulation variation of 1.007-fold was observed in the upstream Hubei Province. The variation in these populations appeared to be associated with their geographic distribution, and the maximum intrapopulation variation increased along the Yangtze River as follows: 1.203-fold in upper and middle reaches Hunan Province, 1.132-fold in upper and middle reaches Hubei Province, 1.238-fold in upper and middle reaches Hubei Province, and 1.436-fold in downstream Jiangsu Province (Fig. 3).

### Geographic association with the 2C nuclear DNA content of *M. lutarioriparius*

Relationships between the relative DNA content (sample mean 2C-values) and geographic variables (longitude and latitude) were investigated using Spearman’s rank correlation. The DNA content was significantly correlated with both latitude (r = 0.697, n = 79, p < 0.01) and longitude (r = 0.685, n = 79, p < 0.01). This result implied that the DNA content increased from south to north and from west to east.

## Discussion

The nuclear DNA content of five species of *Miscanthus (M. lutarioriparius, M. floridulus, M. sacchariflorus, M. sinensis*, and *M.* × *giganteus*) was estimated in this article. The results estimated in our analysis (Supplementary Table S1–2) were comparable to those of previous reports by Rayburn *et al*.^11^, Nishiwaki *et al*.^12^ and Chae *et al*.^10^,^11^,^12^. The mean DNA content of *M. sacchariflorus* was lower than that reported in a previous study (3.96 ± 0.3 pg/2C vs 4.3 ± 0.2 pg/2C). However, the estimates for the other four species were similar to previous reports (*M. lutarioriparius:* 4.26 ± 0.6 pg/2C vs 4.37 ± 0.02 pg/2C; *M. sinensis*: 5.27 ± 0.2 pg/2C vs 5.2 ± 0.2 pg/2C; *M. floridulus:* 5.18 ± 0.3 pg/2C vs 5.1 ± 0.2 pg/2C; *M.* × *giganteus*: 6.93 ± 0.1 pg/2C vs 7.0 pg/2C). This indicates that the estimates in our study are reasonable. The differences in *M. sacchariflorus* might indicate possible genomic diversity in this species.

Multiple comparisons suggested that there was considerable DNA content variation among the five species collected from a range of altitudes and latitudes in China. Moreover, the nuclear DNA content differed between *M. lutarioriparius* and *M. sacchariflorus* by 9.21%, and it differed between *M. lutarioriparius* and *M. sinensis* by 21%. Although a standardized list of morphological descriptors has not been published, the spikelet and inflorescence characteristics have predominantly been used to study variation in *Miscanthus* species^16^. Morphological traits also vary between *M. lutarioriparius* and *M. sacchariflorus* and between *M. lutarioriparius* and *M. sinensis*. The height and diameter of the culms and the numbers of branches on the upper nodes in *M. lutarioriparius* were greater than those in *M. sacchariflorus*^16^. The differences were particularly clear between *M. lutarioriparius* and *M. sinensis* for the inflorescence axis length, raceme length and numbers, spikelet size, spikelet callus hair length, glume and lemma size, dorsal hairs of glume, nerves on glumes, and presence or absence of awns^16^,^17^. The DNA content of *M. floridulus* was similar to that of *M. sinensis* in our results, which was consistent with the results of Hodkinson *et al*.^1^. In their study, *M. floridulus* accessions were embedded in the *M. sinensis* clade. These results may indicate that the DNA content could provide information for taxonomic and evolutionary studies in *Miscanthus* species^10^,^13^. DNA content is important in the evolution and adaptation of plants^18^. Soltis *et al*.^19^ analyzed the evolution of DNA content in angiosperms using a phylogenetic approach and found that it generally increased over time; however, they stated that genome size is dynamic, with both decreases and increases occurring. *Sorghum* is related to *Miscanthus*, and there was highly conserved synteny between the two genomes^20^. Although the evolutionary changes in *Miscanthus* DNA content have not been verified, evolutionary reductions in genome size have been identified in *Sorghum* species^21^. These results suggest that considering the evolutionary relationships of *Miscanthus* in regard to DNA content might offer a new perspective for exploring their different phylogenetic clades. The mechanisms for DNA variation are not clear, but they may be closely related to natural selection to reduce the nucleotypic effects of increased DNA content^22^,^23^,^24^. We speculate that the diverse environments and geographical separation of these four species contributed to their cytological variation.

Genome doubling is an important process in plant evolution, and reports have shown that nearly all vascular plants have undergone at least one round of polyploidy during their evolution^25^,^26^,^27^. *Miscanthus* is a complex polyploid with species that reproduce both sexually and by rhizomes^17^,^28^. Tetraploid accessions were found in *Miscanthus lutarioriparius, M. sacchariflorus*, and *M. sinensis*, which had an average DNA content (2C = 4x = 76) of 8.34 ± 1.2, 8.42, and 8.355 pg, respectively. The monoploid DNA content (1Cx-values) was also significantly different among ploidy levels. From our study, the 1Cx-values for tetraploid *M. lutarioriparius, M. sacchariflorus* and *M. sinensis* were 2.085 ± 0.3, 2.105, and 2.089 ± 0.2 pg, respectively. The 1Cx-values for diploid *M. lutarioriparius, M. sacchariflorus* and *M. sinensis* were 2.128 ± 0.3, 1.978 ± 0.2, and 2.636 ± 0.3 pg, respectively. These results were generally consistent with previous reports, thereby suggesting that diploid and tetraploid *M. sacchariflorus* are taxonomically different; the latter is more closely related to *M. lutarioriparius var. lutarioriparius* than the former is ref. 10. *Miscanthus lutarioriparius* and *M. sinensis* (but not *M. sacchariflorus*) had smaller 1Cx-values in tetraploids than did diploids. These trends of genome downsizing with increasing ploidy levels commonly occurred immediately after the formation of the polyploid^22^,^29^, as in *Ranunculus parnassifolius* L.^30^; *Hieracium bauhinia* Besser^31^; *Cardamine yezoensis* Maxim^32^ and prairie cordgrass^33^. This change may be influenced by the rapid non-random elimination of certain non-coding DNA sequences, the different life cycle strategies of species, or environmental changes^34^. Genome downsizing in the process of polyploidy may increase plant environmental adaptive fitness and facilitate competition with their diploid species. These altered 1Cx-values in *M. sacchariflorus, M. lutarioriparius*, and *M. sinensis* also reflect the plasticity of the polyploid genome in different species.

Correlations between ecological and geographical differentiation and genome size have been reported at both the interspecific and intraspecific levels^35^. Although interspecies genome size differences among related species are widely accepted, intraspecific variation remains controversial^36^. The present study revealed considerable intraspecific DNA content variations in the three species *M. floridulus, M. sacchariflorus*, and *M. sinensis*, but these cannot be automatically assumed to be real because of limited sampling. In previous studies, intraspecific DNA content variability was correlated with geographic environment and ploidy levels in *Festuca pallens*^36^, *Flax*^37^ and *Hordeum spontaneum*^38^. DNA content may affect both the ecological adaptation and distribution^39^. Geographic and ecological parameters such as latitude, temperature, moisture and growth form have been correlated with DNA content in other plants^18^,^39^. Generally, plants that inhabit downstream, moist habitats have larger genomes than do those inhabiting upper reaches with drier habitats^40^,^41^. *Miscanthus lutarioriparius* also had greater nuclear DNA content variation along its distribution range (P < 0.01) in our present study. The samples in this study covered a large region of the natural geographic distribution of this species along the Yangtze River. The genome variation in different accessions along the Yangtze River was compared based on nuclear DNA content. Based on the geographic distribution, the areas of Yangtze River were divided into the following five populations: upstream Hubei Province, upper and middle reaches Hunan Province, upper and middle reaches Hubei Province, middle reaches Anhui Province and downstream Jiangsu Province. Patterns in DNA content variation were found to be associated with the collection sites. Specifically, 0.7% variation was found in upstream Hubei Province, 20.3% in upper and middle reaches Hunan Province, 13.2% in upper and middle reaches Hubei Province, 23.8% in middle reaches Anhui Province, and 43.6% in downstream Jiangsu Province (Fig. 3). In general, the variation rates tended to increase along the Yangtze River. The relationships between relative nuclear DNA content (sample mean values) and geographic variables (longitude and latitude) were investigated using Spearman’s rank correlation, which indicated significant correlations between DNA content and the latitude or longitude. This implied increasing nuclear DNA content from south to north and from west to east. Nuclear DNA content is apparently an important factor that could reflect the evolution and adaptation of plant species^22^,^42^. DNA content can also influence cellular properties such as the nuclear volume, cell volume, the generation of mitosis and meiosis, and generation time^14^,^43^. Previous research^44^ found that genome size variation primarily followed an increasing trend during the evolutionary process. The reasons for the pattern of *M. lutarioriparius* genome size variation have not been determined but may be explained by self-incompatibility, mode of reproduction, mode of seed dispersal, or adaptability to various environments. The seeds of tetraploid *M. lutarioriparius* propagate in water, which might explain the increased genetic variation downstream. The existence of different populations with different DNA content indicates that the pattern observed is complex and may be affected by multiple factors. Future studies employing additional molecular methods may provide the best opportunity for understanding these processes.

Tetraploid accessions were found in *M. lutarioriparius* with an average genome size (2C = 4x = 76) of 8.34 ± 1.2 pg. When we consider ploidy in regard to geographic distribution, a consistent pattern emerged. Almost all tetraploid plants were in well-delimited clusters located in downstream coastal regions. Specifically, these regions spanned from 33°17′12.72″ north, 118°54′37.92″ east to 31°4′21.00″ north, 120°55′39.84″ east and extended from Taihu Lake to Hongze Lake (Fig. 2). Populations in one geographic range showing a single cytotype have been reported in *Dianthus broter*^45^*. Miscanthus lutarioriparius* have wind-pollinated, riparian, and water-logged habitats. The continuity of river and lake systems and the seed dispersal mechanisms may contribute to the higher diversity between different populations and might explain the provincial distribution of tetraploid plants. According to previous studies^46^,^47^, higher ploidy populations might originate in overlapping regions between lower and higher ploidy. In this article, the overlapping regions were in Jiangsu Province (Fig. 2). Such overlaps may occur where the ecosystem or environment changes; for example the overlapping regions might cause the higher ploidy populations to adapt to a lower elevation. The factors that lead to the pattern of ploidy are not clear but might be associated with natural selection and variation in ecological tolerance. Polyploids may have a wider spectrum of tolerance and adaptation to local ecological conditions compared to diploids^47^. Several studies have identified factors that may enhance the fitness of tetraploids in other plants, such as stochastic variation in cytotype frequencies, increased self-fertilization, increased cell size, and an increased rate of cell division^48^,^49^. We envision three scenarios that may explain patterns of different ploidy distributions in *M. lutarioriparius*. First, tetraploid plants mostly cluster in high-latitude regions; patterns of ploidy increasing with latitude have been reported before^50^,^51^. There may be a trend of increasing ploidy from south to north, and tetraploid *M. lutarioriparius* might have a high tolerance for cold and frost. Second, the different distribution between tetraploids and diploids may be related to differences in flowering time. Doubled genomes require more time for cell replication, which can lead to slower cell growth rates and delayed flowering time^14^. The differences in flowering time could lead to the reproductive isolation of new polyploids from their progenitors. Tate *et al*. (2005)^52^ also found that variation in the geographic distribution of diploid and tetraploid accessions of *Anthoxanthum alpinum* appeared to relate to differences in flowering time. Third, tetraploid *M. lutarioriparius* was clustered in areas surrounding lakes along the Yangtze River, and this distribution may indicate rich groundwater resources. The cluster of tetraploids may also be associated with precipitation and submergence^53^.

This study is a comprehensive report on *M. lutarioriparius* collected from various locations throughout China. Their nuclear DNA content and ploidy level were investigated. DNA content is one of the most important parameters that contribute to plant systematics, phylogeny, phylogenetic relationship estimation, and plant adaptation^54^,^55^,^56^. Using comparisons of nuclear DNA content as an adaptive character can shed light on evolution and systematics of narrow taxonomic groups. Geographical isolation and environment affected the DNA content in *M. lutarioriparius*, whereas this phenomenon was not significant in *M. sacchariflorus, M. floridulus*, and *M. sinensis*. These results further strengthened our understanding of the potential geographical genome model of *M. lutarioriparius*, thereby indicating a new perspective for exploring the evolutionary origins of *M. lutarioriparius* or other species. Knowledge of the geographical distribution of various ploidy levels and nuclear content are important not only in the evolutionary study of this species but also in the development of future breeding programs for high yielding and adaptable cultivars. Our study only presented some cytological evidence, but further research is needed to explore the relationship between geographical distribution and evolutionary origins.

## Materials and Methods

### Plant material

Overall, 79 *M. lutarioriparius*, 8 *M. floridulus*, 6 *M. sacchariflorus*, 7 *M. sinensis*, and 4 *M.* × *giganteus* plants were analyzed by flow cytometry. All samples of *M. sacchariflorus* and *M. floridulus* and four samples of *M. sinensis* occurred naturally, and plants were obtained from the middle and lower Yangtze River. A total of 79 *M. lutarioriparius* plants were collected from the reaches of the Yangtze River (Supplementary Table S1), thereby reflecting the native geographical distribution of these species in China. Four clonal individuals of *M.* × *giganteus* were collected from Professor Wu Ju-ying (Beijing Research Development Center for Grass and Environment). The other three *M. sinensis cv., M. sinensis ‘morning light*,*’ M. sinensis Andress ‘Zebrinus’* plants were purchased from market gardens. The seeds of *Oryza sativa* L. spp var Nipponbare (internal standard) were collected from Stake key laboratory of hybrid rice, Wuhan University. All the above-mentioned plants were transplanted in the same germplasm nursery at Wuhan University, China, for 3 or more years.

### Sample preparation and cytometric analysis

For nuclear DNA content determinations, flow cytometric analyses was conducted as described previously^11^,^57^. The internal standard used to quantify DNA was *Oryza sativa* L. spp var Nipponbare with 0.9 pg of 2C nuclear DNA^58^. Briefly, approximately 30 mg of young leaf tissue (expected to contain minimal secondary compounds) of *Miscanthus* and 30 mg of *O. sativa* L. spp. japonica var Nipponbare (internal standard) were used soon after collection. Samples and the internal standard were co-chopped on ice with a sharp razor blade in 1.5 ml of nuclei extraction buffer. The buffer contained 5.55 mg of KCL, 3.69 mg of MgSO_4_, 1.8 mg of HEPES, 36.3 μl of 10% Triton X-100, and a small amount of 5% polyphenol oxidation. The nuclear suspension was filtered through a 30 μm mesh size nylon cloth into a labeled test tube. Following filtration, the supernatant was centrifuged at 3000 rpm at 4 °C for 1.5 min, and nuclei were resuspended in 450 μl MgSO_4_·buffer (5.55 mg of KCL, 3.69 mg of MgSO_4_, 1.8 mg of HEPES). Next, 50 μl of RNase A (50 μg/ml) was added to prevent staining of double-stranded RNA. After resuspension, the mixture was stained with 5 μl propidium iodide (PI) and incubated in the dark at 37 °C for 15 min.

Nuclei were analyzed using a Phoenix Flow Systems flow cytometer (Beckman and Coulter) with an excitation wavelength of 488 nm. A minimum of 10.000 nuclei were analyzed and measured 2 to 3 times per sample. To minimize instrumental drift, replicate measurements were taken on different days and in different individuals. In every sample set, the samples were measured in a random order. Samples in which genome variation was greater than 5% in the three replicates were excluded to eliminate the effects of methodological artifacts. The nuclear DNA content of each sample was calculated using the following formula:


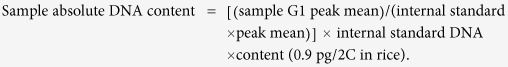


Monoploid genome size (1Cx-value) was estimated as the amount of absolute nuclear DNA content divided by ploidy level.

### Statistical analysis

The interspecific variation of four species was tested by one-way ANOVA. The homogeneity among the genome sizes of *M. lutarioriparius, M. floridulus, M. sacchariflorus*, and *M. sinensis* was evaluated using Student-Newman-Keuls (SNK) multiple comparison tests. Tetraploid data, which may cause false results, were omitted. SNK analysis was also conducted on *M. lutarioriparius* to analyze the differences among five wild populations that represented different geographic distributions. Relationships between relative genome size (sample mean values) and geographic variables (longitude and latitude) were investigated using Spearman’s rank correlation. All statistical analyses were done using the Statistical Product and Service Solutions (SPSS) 8.0 software.

## Additional Information

**How to cite this article**: Sheng, J. *et al*. Nuclear DNA content in Miscanthus sp. and the geographical variation pattern in *Miscanthus lutarioriparius. Sci. Rep.* **6**, 34342; doi: 10.1038/srep34342 (2016).

## Supplementary Material

Supplementary Information

## Figures and Tables

**Table 1:** 2C DNA content homogeneous subsets for the four species Miscanthus by Student-Newman-Keuls analysis.

	Species	N	subsets		
			1	2	3
Student-Newman-Keuls	*M. sacchariflorus*	15	3.9639		
	*M. lutarioriparis*	183		4.2498	
	*M. sinensis*	18			5.272
	*M. floridulus*	24			5.1742
	Significant.		1.000	1.000	.708

From the analyses, the four species were distinct and were divided into three subsets. More specifically, *M. floridulus* and *M. sinensis* were classified together under set 3, whereas the other two species were divided into sets 1 and 2.

**Table 2:** 2C DNA content homogeneous subsets for the four populations *M.lutarioriparius* by Student-Newman-Keuls analysis.

	Populations	N	subsets		
			1	2	3
Student-Newman-Keuls	1. upstream Hubei	5	3.978		
	2. upper and middle reaches Huna	16		4.2506	
	3. upper and middle reaches Hubei	15		4.276	
	4. middle reaches Anhui	24		4.2942	
	5. downstream Jiangsu	21			8.276
	Significant.		1	0.926	1

From the analyses, the four species were distinct and were divided into three subsets. More specifically, *M. floridulus* and *M. sinensis* were classified together under set 3, whereas the other two species were divided into sets 1 and 2.
