# Supplementary material for: Nuclear DNA content in Miscanthus sp. and the geographical variation pattern in Miscanthus lutarioriparius
Source: Sci Rep. 2016 Oct 4;6:34342. doi: 10.1038/srep34342 (PMC5048105; doi:10.1038/srep34342)
Supplement: Supplementary Information [file srep34342-s1.pdf]

Supplementary Information

Nuclear DNA content in Miscanthus sp. and the geographical variation pattern in *Miscanthus lutarioriparius*

Jiajing Sheng[1] Xiaohu Hu[1] Xiaofei Zeng[1] Ye LI[1] Fasong Zhou[1] Zhongli Hu[1] Surong Jin[2]* Ying Diao[1]*

1 State Key Laboratory of Hybrid Rice, College of Life Science, Wuhan University, Wuhan 430072, China;

2 School of Chemistry,Chemical Engineering and Life Sciences, Wuhan University of Technology, Wuhan 430070, China

Correspondence: Ying Diao. Fax: +86-27-68753606, E-mail: ydiao@whu.edu.cn
Surong Jin. Fax: +86-27-68753611, E-mail: jinsr@163.com

Supplementary Table S1 2C DNA content, 1C mean genome size and geographic origin of 79 *M. lutarioriparius* accessions

Accession	2C DNA content	2C/2n	Mean genome size	Latitude	Longitude
name	Mean ±SE		(Mbp/1C)	(N)	(E)
A-03-5	4.23	2.115	2068.47	29°59.359'	113°56.022
A-02-5	4.22	2.11	2063.58	30°07.784'	114°06.835'
A-05-5	4.28	2.14	2092.92	29°32.007'	114°05.832'
A-01-5	4.11	2.055	2009.79	30°07.784'	114°06.835'
A-15-1	4.19	2.095	2048.91	29°37.695'	116°14.253'
A-16-5	4.19	2.095	2048.91	29°43.506'	116°13.797'
B-02-5	4.35	2.175	2127.15	29°44.247'	116°15.285'
B-03-5	4.36	2.18	2132.04	29°46.057'	116°18.558'
B-05-5	4.27	2.135	2088.03	30°01.215'	116°42.225'
B-06-5	4.05	2.025	1980.45	30°01.215'	116°42.225'
B-07-1	4.17	2.085	2039.13	30°08.091'	117°00.814'
B-12-1	4.35	2.175	2127.15	30°30.287'	117°01.599'
B-13-5	4.14	2.07	2024.46	30°28.089'	117°08.870'
B-04-5	4.15	2.075	2029.35	29°46.057'	116°18.558'
B-15-1	4.2	2.1	2053.8	30°28.538'	117°13.170'
B-08-5	4.19	2.095	2048.91	30°03.318'	116°45.704'
C-01-5	4.21	2.105	2058.69	30°45.507'	117°36.995'
C-02-5	4.57	2.285	2234.73	30°45.507'	117°36.995'
C-04-1	4.37	2.183	2134.49	30°54.775'	117°48.169'
C-04-5	4.79	2.395	2342.31	30°46.167'	117°39.501'

C-10-5	4.46	2.23	2180.94	31 °14.938'	118 °18.002'
C-12-1	4.29	2.145	2097.81	31 °33.909'	118 °28.541'
C-12-5	4.39	2.195	2146.71	31 °15.863'	118 °40.692'
C-13-5	4.42	2.21	2161.38	31 °49.285'	118 °34.692'
C-15-1	8.12	4.06	3970.68	32 °08.758'	119 °00.916'
C-16-5	4.12	2.06	2014.68	31 °50.370'	118 °35.668'
C-08-5	4.49	2.245	2195.61	31 °00.756'	118 °03.757'
D-02-5	8.07	4.035	3946.23	32 °10.799'	119 °13.314'
D-04-5	7.76	3.88	3794.64	32 °10.766'	119 °19.409'
D-10-1	4.45	2.225	2176.05	31 °37.676'	120 °04.645'
D-15-1	8.24	4.12	3980.46	31 °04.350'	120 °55.664'
D-16-5	8.48	4.24	4146.72	31 °13.121'	120 °28.516'
D-06-5	8.55	4.275	4180.95	31 °57.571'	119 °49.167'
E-03-5	8.61	4.305	4210.29	32 °20.249'	119 °46.889'
E-04-1	7.71	3.855	3770.19	32 °17.623'	119 °31.015'
E-07-1	8.43	4.215	4122.27	33 °05.709'	118 °34.159'
E-09-5	9.5	4.75	4645.5	33 °04.482'	118 °34.797'
E-01-1	7.45	3.725	3643.05	31 °29.629'	121 °16.407'
E-12-5	8.73	4.365	4268.97	33 °02.535'	118 °37.124'
E-13-1	8.17	4.085	3995.13	33 °02.486'	118 °43.526'
E-13-5	7.81	3.905	3819.09	33 °16.708'	118 °49.765'
E-16-5	8.39	4.195	4102.71	33 °17.212'	118 °54.632'
F-01-5	8.43	4.215	4122.27	33 °06.866'	119 °02.924'
F-04-5	8.42	4.21	4117.38	33 °05.938'	119 °02.476'
F-06-5	8.29	4.145	4053.81	33 °00.922'	118 °58.444'
F-07-5	7.82	3.91	3823.98	33 °00.761'	119 °03.614'
F-14-1	8.01	4.005	3916.89	33 °03.452'	119 °24.163'
F-16-1	4.34	2.17	2122.26	30 °23.522'	114 °07.791'
F-08-1	8.81	4.405	4308.09	33 °04.274'	119 °07.675'
G-01-5	4.41	2.205	2156.49	30 °12.885'	113 °50.326'
G-04-5	4.46	2.23	2180.94	30 °10.505'	113 °51.052'
G-10-5	4.39	2.195	2146.71	29 °37.733'	113 °07.456'
G-11-1	4.51	2.255	2205.39	29 °52.127'	112 °50.242'
G-12-5	4.39	2.195	2146.71	29 °40.649'	113 °06.758'
G-13-5	3.97	1.985	1941.33	30 °04.368'	112 °24.636'
G-02-1	4.47	2.175	2127.15	30 °20.838'	114 °03.446'
G-05-5	4.74	2.37	2317.86	29 °46.540'	113 °24.441'
H-01-1	4	2	1956	30 °13.226'	112 °04.471'
H-01-5	3.98	1.99	1946.22	30 °15.548'	111 °33.107'
H-02-5	3.97	1.985	1941.33	30 °15.548'	111 °33.107'
H-03-5	3.97	1.985	1941.33	30 °24.674'	111 °25.936'
H-04-1	4	2	1956	30 °23.913'	113 °01.310'
H-05-5	8.58	4.29	4195.62	31 °51.022'	113 °03.640'
H-10-5	4.44	2.22	2171.16	31 °27.689'	112 °28.107'

H-11-1	4.44	2.22	2171.16	30°57.414'	112°39.037'
H-11-5	4.28	2.14	2092.92	31°25.850'	112°34.793'
H-14-4	3.7	1.85	1809.3	30°41.296'	112°51.729'
H-16-5	4.3	2.15	2102.7	30°40.772'	112°55.372'
H-05-1	4.31	2.155	2107.59	30°23.913'	113°01.310'
I-10-1	3.93	1.965	1921.77	29°09.283'	112°02.169'
I-11-1	4.25	2.125	2078.25	29°06.640'	111°59.121'
I-13-1	4.25	2.12	2073.36	29°22.919'	111°59.395'
I-14-1	4.25	2.125	2078.25	29°32.274'	112°11.343'
I-14-5	4.25	2.125	2078.25	29°25.496'	112°09.587'
I-15-1	4.21	2.105	2058.69	29°33.360'	112°11.867'
I-03-1	4.17	2.085	2039.13	29°34.991'	113°17.515'
I-04-5	4.1	2.05	2004.9	29°27.698'	112°55.303'
I-07-1	4.36	2.18	2132.04	29°17.930'	112°37.570'
I-09-5	4.12	2.06	2014.68	29°08.192'	112°15.551'

Supplementary Table S2 2C DNA content and 1C mean genome size of 7 *M. sinensis*, 8 *M. floridulus*, 6 *M. sacchariflorus*, and 4 *M. x giganteous* accessions.

Accession name	2C DNA content Mean ±SE	2C/2n	Mean genome size (Mbp/1C)
M. sinensis			
mg108	8.52	4.26	4166.28
mg114	5.39	2.695	2635.71
mg111	5.42	2.71	2650.38
mg001	5.1	2.55	2493.9
mg040	5.24	2.62	2562.36
mg002	5.14	2.57	2513.46
mg165	5.37	2.685	2625.93
M. floridulus			
mg113	5.06	2.53	2474.34
mg147	5.38	2.69	2630.82
mg150	5.32	2.66	2601.48
mg152	4.9	2.45	2396.1
mg156	5.08	2.54	2484.12
mg157	5.06	2.53	2474.34
mg101	5.1	2.55	2493.9
mg110	5.5	2.75	2689.5
M. sacchariflorus			
mg116	3.83	1.915	1872.87
mg129	4.14	2.07	2024.46
mg136	3.76	1.88	1838.64
mg137	3.98	1.99	1946.22
mg138	4.07	2.035	1990.23
mg063	8.42	4.21	4117.38
M. x giganteous			
mg0244	6.97	3.485	3408.33
mgo245	6.97	3.485	3408.33
mg0249	6.95	3.475	3398.55
mg0250	6.84	3.42	3344.76
